# “We’re always an afterthought”- Designing tobacco control campaigns for dissemination with and to LGBTQ +—serving community organizations: a thematic analysis

**DOI:** 10.1007/s10552-023-01706-x

**Published:** 2023-05-09

**Authors:** Shoba Ramanadhan, Meg Salvia, Elaine Hanby, Anna C. Revette, Madison K. Rivard, N. F. N. Scout, Julia Applegate, Bob Gordon, Ana Machado, Mitchell R. Lunn, Juno Obedin-Maliver, Jennifer Potter, Jarvis T. Chen, Andy S. L. Tan

**Affiliations:** 1 Harvard T.H. Chan School of Public Health, Boston, MA USA; 2 Annenberg School for Communication, University of Pennsylvania, Philadelphia, PA USA; 3 Dana-Farber Cancer Institute, Boston, MA USA; 4 LGBT Cancer Network, Providence, RI USA; 5 Ohio State University, Columbus, OH USA; 6 California LGBT Tobacco Education Partnership, San Francisco, CA USA; 7 CenterLink, Inc, Fort Lauderdale, FL USA; 8 Stanford University School of Medicine, Palo Alto, CA USA; 9 Fenway Health, Boston, MA USA; 10 Harvard Medical School, Boston, USA

**Keywords:** Dissemination, LGBTQ, Tobacco, Health campaign, Community-based organizations, Participatory research

## Abstract

**Purpose:**

Evidence-based health communication campaigns can support tobacco control and address tobacco-related inequities among lesbian, gay, bisexual, transgender, and queer (LGBTQ +) populations. Community organizations focused on LGBTQ + health (e.g., nonprofits, community centers, and community health centers) can be prime channels for delivering evidence-based health communication campaigns. However, it is unclear how to balance the goals of a) designing campaigns to support broad adoption/uptake and b) adaptation addressing the needs of diverse communities and contexts. As part of an effort to support “designing for dissemination,” we explored the key challenges and opportunities staff and leaders of LGBTQ + -serving community organizations encounter when adopting or adapting evidence-based health communication campaigns.

**Methods:**

A team of researchers and advisory committee members conducted this study, many of whom have lived, research, and/or practice experience with LGBTQ + health. We interviewed 22 staff members and leaders of community organizations serving LGBTQ + populations in the US in early 2021. We used a team-based, reflexive thematic analysis approach.

**Results:**

The findings highlight the challenges of attempting to use health communication campaigns misaligned with the assets and needs of organizations and community members. The three major themes identified were as follows: (1) available evidence-based health communication campaigns typically do not sufficiently center LGBTQ + communities, (2) negotiation regarding campaign utilization places additional burden on practitioners who have to act as “gatekeepers,” and (3) processes of using health communication campaigns often conflict with organizational efforts to engage community members in adoption and adaptation activities.

**Conclusions:**

We offer a set of considerations to support collaborative design and dissemination of health communication campaigns to organizations serving LGBTQ + communities: (1) develop campaigns with and for LGBTQ + populations, (2) attend to the broader structural forces impacting campaign recipients, (3) support in-house testing and adaptations, and (4) increase access to granular data for community organizations.

## Background

Evidence-based health communication campaigns (e.g., television advertisements, radio broadcasts, print materials, and digital messaging) can effectively change tobacco-related knowledge, attitudes, and beliefs and prevent tobacco initiation or support tobacco reduction and cessation [1]. We focus on tobacco control among lesbian, gay, bisexual, transgender, and queer (LGBTQ +) populations in the US, who have higher rates of tobacco use than non-LGBTQ + populations [2, 3]. These inequities may result from targeted marketing by the tobacco industry, minority stress, alcohol and/or substance co-use, and reduced access to healthcare services [4–9]. Community organizations such as nonprofits, community centers, and community health centers are prime partners for adopting and adapting evidence-based health communication campaigns for delivery among LGBTQ + communities, given their reach and trust among these groups [10–12]. They have tremendous potential for scale; a nationally representative survey found that 19% of nonprofits explicitly focus on LGBTQ + people [13] and more than 250 LGBTQ + community centers provide community-building, educational, and health services [14, 15]. Despite the availability of potential partners, it is not a simple task for campaign developers and community organizations to connect regarding adopting and adapting evidence-based health communication campaigns. Two barriers exist to the widespread delivery of effective campaigns. First, there is a limited evidence base of tobacco control campaigns for LGBTQ + individuals [16, 17]. Second, it is unclear how to balance goals of (a) widespread dissemination of evidence-based cancer prevention and control campaigns that lead to adoption/uptake and (b) supporting adaptation to meet the needs of diverse communities and organizations [18].

A “designing for dissemination and sustainability” lens can improve the fit between the evidence base and the needs of community organizations focused on LGBTQ + health. This perspective on evidence generation centers the resources, requirements, and context of individuals and organizations adopting and adapting programs [18–20]. As part of an effort to reimagine supports for adopting and adapting evidence-based health communication campaigns, we gathered qualitative data from staff and leaders of LGBTQ + -serving community organizations in the US. Our goal was to identify key factors that hinder the ability of LGBTQ + -serving community organizations to adopt or adapt evidence-based health communication campaigns addressing tobacco use.

## Methods

### Parent project

Data for this study come from Project Resist, which examines the effects of culturally tailored messages on resistance to tobacco industry marketing among young adult sexual minority women. The focus on this population reflects the fact that women aged 18–24 in the US who identify as lesbian, gay, bisexual, or a member of another sexual minority group have up to 4.8 times the odds of regular cigarette smoking than their heterosexual counterparts. (We note that this statistic is based on data limited to binary female/male categories) [21]. The emphasis on industry marketing reflects a growing evidence base suggesting that campaigns highlighting tobacco companies’ targeted marketing to LGBTQ + people have increased resistance to such tactics [22–24]. While Project Resist focuses on young adult sexual minority women, this inquiry also included a focus on LGBTQ + health more broadly.

### Study design and team composition

We conducted semi-structured interviews with practitioners in community organizations addressing LGBTQ + health. We approached the work with critical and constructivist perspectives, understanding that the knowledge from this study (a) would be co-created by the study team, the external advisory committee, and community organization practitioners in a reflection of our values and positions and (b) is generated with a commitment to addressing injustice through transformative processes [25, 26] The analysis team included individuals with expertise in LGBTQ + health, implementation science, health communication, community delivery of health services, qualitative methods, and cancer inequities. One analysis team member brought lived experience as a member of the LGBTQ + community. All analysis team members emphasize social justice and health equity in their work and attempted to be explicit about how the boundaries of their knowledge impacted the analysis. The team also drew on the expertise of a larger group of academic researchers with lived and/or research experience with LGBTQ + health. Additionally, the project uses a consultation model of participatory research [27] to engage four nationally recognized leaders in the area of LGBTQ + health as an external advisory committee. For this study, they offered an orientation to community organizations’ use of research evidence, reviewed and revised the interview guide, facilitated connections for recruitment, and supported the interpretation of results. All four are co-authors on this manuscript. The core analysis team and external advisory committee include members for whom intersectionality (or the intersection of multiply marginalized identities) is part of their lived experience and/or professional focus.

### Participants

We interviewed individuals who direct or run health promotion in community organizations (nonprofits, community centers, and community health centers) serving LGBTQ + populations in the United States (*n* = 22). We sought individuals knowledgeable about the organization’s health promotion programs and tobacco control activities, if relevant. Our sampling method included purposeful and snowball approaches. We started with a referral recruitment strategy, connecting with practitioners identified by the advisory committee and interview participants. We also attempted to recruit from a national directory of LGBTQ +—serving organizations (www.lgbthealthlink.org). Our purposeful sampling strategy focused on attaining sufficient information-rich cases [28] among participating organizations regarding the area of action, US region, and LGBTQ + populations served (Table 1).

**Table 1:** Characteristics of practitioners’ organizations, in order of decreasing frequency (*n* = 22)

	Frequencies
Area of action (multiple selections permitted)	
Program delivery	18
Community organizations	12
Health centers (service providers)	12
Advocacy	11
Policy	4
Region	
Southeast	5
West	5
Midwest	4
Northeast	4
Southwest	4
Populations served (multiple selections permitted)	
Young adults	22
All LGBTQ + identities	21
Adolescents	20
Older adults	18

### Data collection

The analysis team used an iterative process to design the semi-structured guide with support from the advisory committee. Interviews opened with questions about participant background and organizational context. To ground our conversation about participants’ experience with tobacco/health outreach, we described examples of evidence-based programs that were generally targeted, such as the Truth Initiative’s campaigns [29], and those that were focused on LGBTQ + populations, such as “This Free Life [30].” We also referenced popular campaigns designed for LGBTQ + populations, such as “When Did Smoking Become Part of Us?” [31]. We did not present examples of materials from these campaigns or ask for responses to these specific campaigns, but instead used them as examples of the types of campaigns we wished to discuss. We then narrowed the focus of the conversation to focus on adopting and adapting the Project Resist campaign. The campaign was described as a health communication campaign for tobacco control that was undergoing testing at the time among young adult sexual minority women. We described the campaign’s focus on building resistance to tobacco industry marketing tactics that target LGBTQ + people.

Interviews took approximately 45 min and were conducted using a videoconference platform between January and June 2021. We explained study procedures and obtained informed consent before beginning the discussions. Interviewers included experienced qualitative researchers with doctoral degrees in public health and social and cultural psychology (SR and JR) and graduate degrees in nutrition (MS) and health communication (EH). The interviewers and participants were unknown to each other before the interview. Participants received $50 gift cards as a token of appreciation. Interviews were audio-recorded and professionally transcribed. The Institutional Review Board at Harvard University approved this study as exempt from review.

### Data analysis

We employed a team-based, reflexive thematic analysis approach. Immediately after each interview, team members independently recorded notes using a prefigured interview summary table. This allowed the team to capture key insights and incorporate participant feedback to inform further data collection. The initial codebook followed the semi-structured interview guide and included deductive codes based on the guide and inductive codes identified from the transcripts and regular analysis meetings. Four transcripts were coded utilizing the initial codebook, and then, the team met to review, modify, and finalize the codebook. At that point, two coders (MS and EA) independently coded the transcripts and systematically resolved discrepancies in coding. We used Nvivo to manage the dataset [32]. After coding was complete, members of the study team summarized selected codes of interest for this analysis. The analysis team (MS, SR, AR, EA, AT) engaged in biweekly meetings to review these summaries and identify connections between codes, develop themes, and select representative quotes.

## Results

This study explored the challenges related to adopting and adapting evidence-based health communication campaigns raised by practitioners from LGBTQ + -serving community organizations. Participants offered commentary based on a diverse set of campaigns they and their organizations had seen, reviewed, and/or implemented. Three major themes were identified: (1) insufficient centering of LGBTQ + populations in many available evidence-based health communication campaigns, (2) negotiation regarding campaign utilization places extra burden on practitioners who have to act as “gatekeepers,” and (3) processes of using health communication campaigns often conflict with organizational efforts to engage community members in adoption and adaptation activities. Overall, the findings highlight the challenges of using campaigns misaligned with the assets and needs of organizations and community members. The results also emphasize the importance of trustworthiness, cultural humility, inclusivity, partnerships, and community engagement as critical (and mutually reinforcing) components of successful programs and campaigns for LGBTQ + populations.

### Theme 1. Available evidence-based health communication campaigns typically do not sufficiently center LGBTQ + communities

Participants expressed concern that LGBTQ + communities were insufficiently centered in the development and dissemination of many broadly available evidence-based campaigns. They described accessing a diverse range of evidence-based campaigns through national and state resources, as well as being provided with these materials by funders. The theme had three dimensions: (1) insufficient inclusiveness of language and materials, (2) lack of consideration of client/community member context, and (3) limited data specific to their populations or service areas.

#### For many broadly available programs, campaign language, materials, and processes are not sufficiently inclusive

An important challenge was the poor centering of LGBTQ + populations in the language and imagery utilized in many campaign materials available to community organizations. This was discussed related to LGBTQ + populations generally and specific LGBTQ + communities. Many participants suggested that LGBTQ + people were not well represented visually or through messaging in existing campaigns.

“It’s just we’re always an afterthought. Let’s just say that. We’re always an afterthought.” – KII #13

“What we did after reviewing those [campaigns] is just determine that none of those campaigns were featuring language and images that would really resonate with queer folks. So if you have a bunch of happy, heteronormative, straight couples running through fields of daisies with their kids—I love my straight friends, but I'm not sure that's going to necessarily speak to the queer community.” – KII #7.

There was consensus about the need for the campaign visuals to be inclusive and represent a diversity of bodies and identities. Criticism of many of the existing materials included an overreliance on stock images and representation of white, presumably cisgender individuals in images. Participants noted that having diverse images represented in the campaign would support their ability to deliver the campaign to a broader audience. Some participants reflected that the materials and campaign content must be explicitly consistent with the organization’s mission. A mismatch between populations served and campaign components made many of the existing campaigns a poor choice for these organizations.

“Even if that’s not a conscious thought – subconsciously, we’re like, oh, that’s for them. It’s not for me, because I'm different than that.”—KII #21

“One of the first things we look for is we see any project that’s looking at just women or men is how inclusive is that of all people and all bodies? And so, that would be one thing that we as an organization would be critical of. If we’re sharing it with our supporters, if we’re in any way kinda backing it, we wanna make sure that the language is very inclusive.” – KII #4.

#### Campaigns typically do not account for the broader contexts of community members’ lives

Another dimension of insufficient centering of LGBTQ + communities was the disconnect between the campaigns and the broader context in which they will be used. Disconnects were often framed as a need for cultural humility on the part of the teams developing campaign materials, particularly given the challenges many LGBTQ + community members are facing. Some participants noted that many LGBTQ + community members are processing trauma or other concerns and thus may be using tobacco as a form of coping or self-medication. In such cases, campaigns emphasizing knowledge regarding the harms of tobacco use were not expected to have high impact. Participants highlighted an opportunity to take a harm reduction frame and support health-promoting behaviors (e.g., reduction of tobacco use) while acknowledging the broader context of tobacco use. A related concern emphasized the need to take a structural focus.

“Let’s meet people’s basic needs… If you can meet folk, and there’s safety, and security, and love, and belonging, and all these other things – we know they’re less likely to utilize substances or do other things that we can deem as at-risk behavior… We can reduce risk by providing for people’s basic needs.” – KII #1.

Finally, participants highlighted several factors that would prompt a need for adaptation, such as demographic attributes (such as race or ethnicity), cultural background, socioeconomic status, and levels of anti-LGBTQ + stigma in their local area. This also connected to discussions that there is not just one “LGBTQ + community” but multiple overlapping LGBTQ + communities.

#### Community organizations need more population-specific data to leverage campaigns

Participants voiced two types of concerns related to insufficient information. First, participants were concerned about the limited availability of granular data broken out by sexual orientation, gender identity, and other attributes. These concerns applied to statistics describing need (e.g., tobacco use rates) and effectiveness data (e.g., the impact of campaign messages on different LGBTQ + communities).

“If it’s really effective in 19-year-old bisexual women, that doesn’t really mean it’s gonna have much impact in a 24-year-old cis gay white man [laughter]. So, I would think we would want some more broad feedback on the acceptability of a message before we make it broadly available.”—KII #3.

Several participants noted that it would not be possible to use national statistics, which are already limited, and apply them to the communities they serve. One participant also stated that they could not access the needed statistics due to journal paywalls. These challenges were linked to difficulties making programming decisions.

For our population in [state]…it’s difficult for us to use national data because of our specific population being a lot of indigenous people and just often having different needs because of our geography. – KII #2.

### Theme 2. Negotiation regarding campaign utilization places extra burden on practitioners who have to act as “gatekeepers”

Participants reported needing to play gatekeeping roles to preserve the safety of community members and maintain trust between community members and their organizations. They described evaluating how academic researchers and campaign materials did or did not communicate respect for LGBTQ + populations. These evaluations influenced whether participants were willing to pursue potential adoption or partnership.

Participants described the intense efforts needed to build trust between the organization and the populations they serve. They described actively vetting potential collaborators and campaigns for cultural humility to ensure programs would benefit their communities and would not cause harm or damage trust. Similarly, they reported actively screening proposed communication campaign materials (and potential partnerships) to assess the presence of harmful or exclusionary content, microaggressions, or content that did not reflect the diversity of the clients and community members they served. Protection against further harm was emphasized for community members holding multiple marginalized identities. While participants described the burden of “gatekeeping” activities, they also noted that these efforts were closely connected to their personal and organizational missions and goals of fostering safe spaces while using resources effectively.

“I think when we are approached for new partnerships, whether that’s research, or messaging, or just project collaboration, we really like to be treated like the experts in the room when it comes to the LGBTQ community because I think we have to sometimes act like the gatekeepers. We’re not just gonna let anyone come into our community and tell our patients what’s up [laughter]. And I think the most – the easiest way to identify that early is just vocabulary.” – KII #3.

“How queer-friendly is your program? It’s like, oh, well, we don't need to be really queer-friendly. Smoking’s a problem for everyone. And I was like, yes, and that’s totally the wrong approach, right?… It’s always kind of like these subtle micro-aggressions that happen.” – KII #22.

“I do believe, with professionals, there’s sometimes just this arrogance, right? And this is true with behavioral health clinicians. I know best, I am going to tell you what to do because I have 18 letters behind my name and therefore you're going to listen to me, and this is going to solve your problems.” – KII #7.

Participants reported assessing campaigns in terms of the value placed on community expertise. A clear indication that campaign developers and materials displayed cultural responsiveness was prioritized over the related evidence base. Similarly, evidence of engagement of LGBTQ + communities during campaign development was highlighted as a valued campaign attribute. This did not negate the need for the organization to consult community members themselves, but it did encourage the organization to consider the campaign more seriously.

“How intentional they [the researchers] are around wanting to be inclusive and also respect the expertise of the community that they’re wanting to serve. Because we may not all be PhD researchers or doctors or CEOs, but we are experts in our own unique lived experience.” KII #22.

### Theme 3. Processes of using health communication campaigns conflict with organizational efforts to engage community members in adoption and adaptation activities

Participants described the importance their institutions place on collaborating with and recognizing the expertise of LGBTQ + community members regarding their health. In the context of health communication campaigns, they also expressed frustration that academic researchers did not seem to respect the expertise and knowledge of community organizations and engage with them as partners. Participants also raised the issue that their organizations are unsupported to lead campaign adaptation and evaluation.

Many community organizations connected with community members via advisory boards. This was particularly important for programs serving young people, who were unlikely to be on staff at the organization. Other organizations used informal advisory boards or peer educators to link program staff and clients/community members. Participants described the balance they sought to achieve between what academic researchers presented as evidence and what their local experts offered. They emphasized that research evidence was one of many factors considered.

“I have to kind of balance what they [the researchers] say is evidence based with what my cultural experts are saying is gonna work for that population, and they don’t always match up.”—KII #2

Participants also highlighted the importance of piloting campaigns and collecting preliminary data to support adaptation, although they noted that lower-budget organizations may not have the resources to conduct those tests.

As a contrast to examples of tensions with campaign developers and distributors, some organizations were able to share implementation experiences as part of an effort to improve campaigns. A larger organization described running a campaign and sharing their findings with the original campaign designer to support iteration.

“A real bedrock of what we do, engaging the stakeholders who are the recipients of those things. How did it work for you? What did you think? What worked great? What didn’t work so well? What suggestions would you make? And then, in a partnership, to be able to come back and be like, so these people said this, these folks had these comments, this was also something that was noted. To be able to share that with [the designer/researcher].” KII #5.

## Discussion

This study explored experiences with adopting and adapting evidence-based health communication campaigns among practitioners from community organizations serving LGBTQ + populations. We identified three pain points for these organizations: (1) insufficient centering of LGBTQ + populations by many broadly disseminated evidence-based campaigns, (2) burden placed on practitioners as they serve as gatekeepers to protect their community members from harm and preserve the trust they have built, and (3) a lack of support for the diverse ways community organizations engage community members while using evidence-based campaigns. These issues highlight opportunities for academic researchers, government agencies, foundations, and other research producers to increase the relevance and utility of the evidence they are producing. Some challenges are easily addressable in the short term, and others are worthy long-term goals. Broadly, opportunities raised include developing campaigns with and for LGBTQ + populations and the organizations that serve them and providing necessary supports (e.g., adaptable messages with many flexible options) for practitioners to select, adapt, and pilot campaigns with limited burden. In the context of this work, a truly partnered approach might include academic researchers offering data and expertise about message creation and CBOs bringing leadership and community-specific knowledge to support customization and localization, with the former being offered in service of the latter.

Opportunity #1: Developing campaigns with and for LGBTQ + populations and the organizations that serve them

Participants expressed frustration as they described the ways many of the campaigns they were offered were not developed for and with the communities they serve. While there are examples of evidence-based tobacco control campaigns developed specifically for LGBTQ + audiences (e.g., [33]), these are still rare at this time. Participants emphasized the opportunity for research producers to engage adopting organizations and LGBTQ + populations in developing campaigns – not as sources of information but as expert advisors or partners. As highlighted in the participatory research literature, such partnerships increase the relevance and credibility of the research and improve the likelihood the evidence will be applied in practice [34]. These and other benefits are highlighted in models of *participatory implementation science,* which promote iterative and ongoing engagement among academic researchers, practitioners, community members, and other partners to support the integration of research evidence into practice. These efforts can address health inequities in the short term through service delivery and in the long term through systems change. A range of engagement levels is available, depending on needs, resources, and goals [27, 35]. A core attribute of participatory approaches is the assets focus [36], which reflects the emphasis participants placed on expertise held by community members and the organizations that serve them. Such an approach connects with the emphasis participants placed on trustworthiness, cultural humility, inclusivity, and partnerships for the successful dissemination of evidence-based campaigns.

Additionally, the need to center LGBTQ + populations in campaign development connects with the literature on culturally tailored materials. The literature is mixed regarding whether or not tailored interventions have greater tobacco control outcomes over non-tailored ones, as highlighted by reviews conducted in 2014 and 2017 [16, 37]. Yet, that literature focuses on individual-level attributes, with little examination of how interpersonal, community, and organizational environments may also drive a need for tailoring. The rationale for culturally tailored health communication campaigns stems from evidence that LGBTQ + community members prefer information that is LGBTQ + specific, inclusive, relatable, and highlights diversity [38]. Health campaigns that do not address the unique needs of LGBTQ + audiences may be ignored and lead to communication inequalities such as lower attention, information processing, and ability to act on health promotion messages. Ultimately, these communication inequalities may widen health inequities [39]. Mismatched materials have resulted in community members perceiving the sponsoring organization as lacking understanding of their needs and losing trust in the institution [40, 41]. Finally, an important takeaway was the emphasis participants placed on campaigns needing to account for the broader context of smokers’ lives, e.g., tobacco use as a response to structural harms, particularly for people holding multiple marginalized identities [42]. In this way, participants’ perspectives resonated with the broader literature emphasizing harm reduction frames as a compassionate response to understanding the structural drivers of tobacco use for many LGBTQ + people [4, 14, 43, 44].

Opportunity #2: Improving supports for community organizations as they assess, adapt, and pilot campaigns

Another critical opportunity lies in increasing the quantity and quality of scalable supports provided to community organizations as they adapt and pilot campaigns. Support may include fiscal resources, investments in staff capacity and networks, and sharing of material resources to facilitate action in community settings [45]. Another set of supports relates to the process of campaign use, as participants consistently described the “heavy lift” of needing to adapt and customize materials for their clientele. Given the emphasis placed on community engagement throughout the community organizations’ process, supports to help programs identify and make needed adaptations, detailed descriptions of the initial participants (and their contexts), and local data to support adaptation may all reduce the burden on organizations and increase the likelihood of adoption and implementation. An excellent example of such resources comes from the MPowerment HIV prevention program for young gay and bisexual men, which offers focus group guides, evaluation materials, and adaptation supports to facilitate the customization by local leaders of the campaign for their communities’ needs [46]. Additionally, given how identities and experiences vary and evolve, the goal should not be for academic researchers to attempt to anticipate all possible needs but instead to support those with local expertise in conducting those activities [47]. This allows for scalable supports, such as implementation manuals and adaptation planning materials, which are critical for achieving population health impacts. Additionally, it can be useful to attend to communication infrastructure that bridges the information environment and communication and health outcomes. Designing health campaigns in a way that is integrated with existing local communication channels and networks can improve information flow, sense-making, and organizing within each community, thereby supporting the advancement of health equity among LGBTQ + populations [48].

Practitioners also highlighted the need for local data to support the selection and adaptation of campaigns. This is particularly important given that the communities they serve are heterogeneous on a range of dimensions. Given that tobacco use varies widely based on LGBTQ + identity, race, ethnicity, socioeconomic status, and other intersecting factors, service organizations need granular data [49]. Recent work with LGBTQ + -serving organizations addressing mental health needs highlighted the importance of addressing intersectionality and increasing the focus on LGBTQ + people of color and less-studied sexual and gender minority groups [14].

**Fig. 1:**
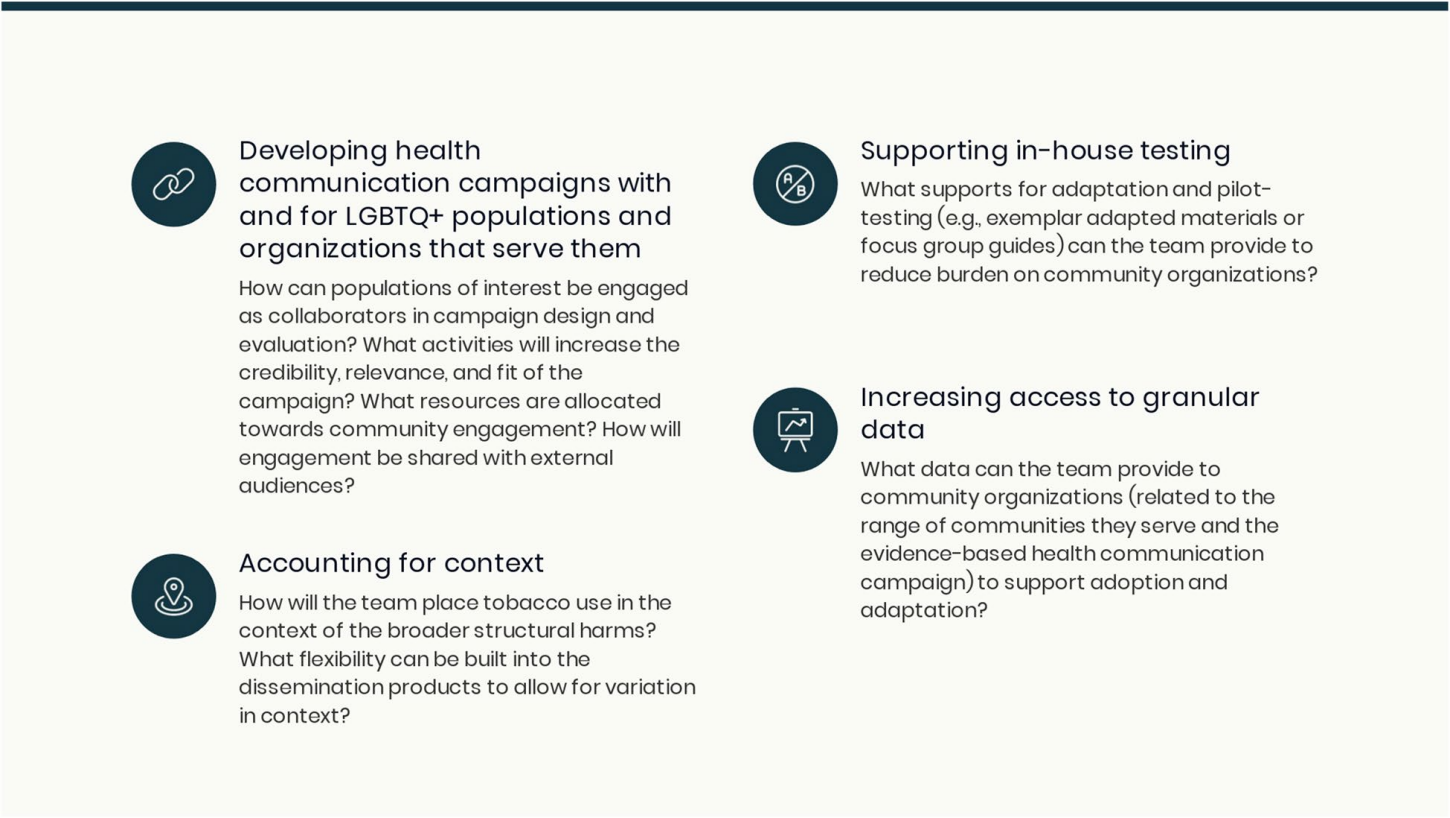
Questions to support collaborative design and dissemination of tobacco-focused health campaigns for use by organizations serving LGBTQ + communities. Caption: LGBTQ + : lesbian, gay, bisexual, transgender, and queer

For research producers who are new to developing campaigns for relevance and impact among organizations serving LGBTQ + populations,, we offer a series of considerations prompted by these results (Fig. 1). The list echoes syntheses of the dissemination literature, which prompt academic researchers to understand what motivates potential adopters; explore differences in perceptions between academic researchers and practitioners; invest in dissemination supports before offering campaigns to practitioners; understand that authority does not equate with influence; leverage the power of change agents; and offer customizable choices [20, 50]. While many of the considerations and offerings from the literature emphasize partnership between academic and practice audiences, we recognize that this is not a standard approach for all researchers. For researchers new to engaged approaches, one challenge in working with marginalized groups is establishing trust and credibility in the focal community, due to historical and current systems that drive inequities [51]. Without credibility in a community and without considering barriers to participation, research teams cannot effectively engage and collaborate with organizations addressing inequities. The rich body of scholarship around community-engaged research and community-based participatory research offers tools for engaging in a trustworthy fashion, including sharing power, demonstrating cultural humility, investing in long-term relationships, and focusing on positive outcomes of importance to diverse partners [35, 52].

We place our findings in the context of a set of limitations. First, we asked participants to respond to an example about a health communication campaign addressing the needs of young adult sexual minority women, but the bulk of the conversations related to LGBTQ + health broadly. This may have limited participants’ ability to offer detailed critiques, but the next step in our research will continue this line of inquiry with prototype campaign materials. Additionally, the use of Project Resist as an exemplar campaign was expected to increase participant engagement and improve the quality of the data collected [53]. Second, we framed our questions in the context of evidence-based public health, which emphasizes inclusion of diverse expertise and preferences [54], but recognize that evidence-based solutions are often deployed in a technocratic manner, devaluing expertise from individuals outside the academy. Finally, perspectives of organizations serving some LGBTQ + populations were not included in the study. At the same time, the study offers important strengths. Notably, we used a participatory approach to support collaborative study design and analysis by a group of community leaders and academic researchers, including a substantial proportion with lived, research, and/or practice experience with LGBTQ + health. Second, the purposeful sampling approach allowed the team to gather data from organizations serving a diversity of LGBTQ + communities across the US. Finally, the rigor of the design and conduct of this study increases the credibility and transferability of results to other “design for dissemination” efforts. Future research should engage staff and leaders of community organizations to co-develop solutions to the challenges identified here. Additional work is also needed to understand how these findings apply beyond tobacco control health communication campaigns.

## Conclusion

This study highlights the importance of an inclusive approach to designing for dissemination that emphasizes community organizations and community strengths. The findings also suggest that designing for dissemination approaches may decrease the burden of gatekeeping and processes of selecting, adapting, and piloting health communication campaigns. For academics, this approach may offer a way to increase the quality and relevance of the tobacco control health communication campaigns produced and their potential reach and impact. In other words, an inclusive, partnered approach increases the ability of researchers to ensure that LGBTQ + populations and the organizations that serve them are co-creators of vital, high-impact solutions, not an afterthought.

## Data Availability

The datasets generated during and/or analyzed during the current study are not publicly available due to privacy concerns, given the small number of organizations working in this space. A redacted version of the dataset will be made available based on requests to the corresponding author.
